# Community-acquired and healthcare-associated *Staphylococcus aureus* infections in a pediatric hospital in southern Brazil over 8 years: how common is MRSA?

**DOI:** 10.3389/fped.2023.1212239

**Published:** 2023-06-12

**Authors:** Derrick Alexandre Fassbind, Raíssa Queiroz Rezende, Cícero Armídio Gomes Dias, Fabrizio Motta

**Affiliations:** ^1^ Pediatric Infectious Disease Department, Santo Antônio Children Hospital, Porto Alegre, Brazil; ^2^ Pediatric Intensive Care Unit, Santo Antônio Children Hospital, Porto Alegre, Brazil; ^3^ Department of Microbiology, Federal University of Health Sciences of Porto Alegre, Porto Alegre, Brazil

**Keywords:** *Staphylococcus aureus*, MRSA - methicillin-resistant *Staphylococcus aureus*, community-acquired MRSA (CA-MRSA), healthcare-related infections, children

## Abstract

**Backgrounds:**

Both healthcare-associated and community-acquired methicillin-resistant *Staphylococcus aureus* (MRSA) infections are relevant in children. The objective of our study was to evaluate their impact in a pediatric hospital in southern Brazil.

**Methods:**

Data from patients under 18 years of age with *S. aureus* infections between January 2013 and December 2020 were retrospectively analyzed. Data were collected regarding infection site, infection type (community-acquired or healthcare-associated), susceptibility to oxacillin [methicillin-susceptible *S. aureus* (MSSA) or MRSA] and other antimicrobials. We analyzed the evolution of the susceptibility rates for the isolates over this period.

**Results:**

A total of 563 patients were included, among whom the prevalences of community- and hospital-acquired MRSA infections were 46.1% and 8.1%, respectively. No significant change occurred in these prevalences over the study period. In community-acquired infections, MSSA was significantly more associated with osteoarticular infections and MRSA was more associated with respiratory and intra-abdominal infections. In healthcare-associated infections, there was an association between MSSA and primary bloodstream infections and between MRSA, skin/soft tissue infections, and respiratory infections. Community-acquired MRSA were highly susceptible to trimethoprim-sulfamethoxazole (96.1%), clindamycin (88.4%), and doxycycline (99.0%).

**Conclusion:**

Our study draws attention to the high rates of MRSA in community-acquired staphylococcal infections in this population, indicating a need to review initial protocols for severe staphylococcal infections according to local epidemiology.

## Introduction

*Staphylococcus aureus* is responsible for a considerable portion of community-acquired (CA) bacterial infections that lead to hospitalization in children and causes several clinical syndromes, including healthcare-associated (HA) infections. Determining the bacterium's susceptibility to oxacillin is the first step in deciding upon a course of treatment; strains susceptible to this antimicrobial are called methicillin/oxacillin-susceptible *S. aureus* (MSSA) or methicillin/oxacillin-resistant *S. aureus* (MRSA).

Although MRSA clones have been historically associated with HA-MRSA, the proportion of clones responsible for community-acquired infections (CA-MRSA) has been increasing significantly worldwide. This epidemiological change has relevant clinical implications, since empirical antibiotic therapy for community-acquired staphylococcal infections often involves beta-lactams, a class that, except for ceftaroline, is not active against MRSA. Although CA-MRSA strains tend to be susceptible to non-beta-lactam antibiotics, the incidence and antimicrobial susceptibility profile can vary greatly and, in Brazil, the subject is little investigated, especially in pediatric populations (1–6).

The present study aims to define the proportion of MRSA isolates in a pediatric hospital in southern Brazil, comparing the characteristics of patients with MRSA and MSSA infections, as well as describing the susceptibility of community-acquired isolates to other antimicrobials.

## Materials and methods

### Design

This retrospective cohort study analyzed the medical records of patients under 18 years of age with an *S. aureus* infection between January 2013 and December 2020 who were treated at the Hospital da Criança Santo Antônio, a tertiary care center and one of the largest pediatric hospitals in southern Brazil, with 200 beds. Brazilian population is covered by a universal healthcare system, provided by the government and free of charge, which allows the population to be treated in public hospitals and clinics. A part of the population also has private and paid health insurance, and can be attended in private clinics and hospitals. As a philanthropic private hospital, this hospital treats children without restrictions, both from the public healthcare system, at no cost, and children who have private health insurance.

### Patient inclusion

All first consecutive positive cultures (collected aseptically) from sterile sites were considered: pleural, peritoneal, joint, or cerebrospinal fluid; abscess, bone, or wound secretion; blood; and urine. Patients in whom *S. aureus* was isolated from other materials (e.g., sputum, tracheal or eye secretion, and open wound or nasal swab) were not included. Samples without clinical relevance (e.g., colonization/contamination when the culture was positive but the patient did not show clinical signs of infection and had a favorable outcome without the use of antibiotic therapy, polymicrobial growth when more than one pathogen was isolated from the same culture, or those missing clinical information in the medical records) were excluded after authors' assessment.

### Definitions

Infections were considered CA when the following criteria were present: signs and symptoms of infection prior to hospitalization and culture obtained within 48 h of hospitalization, no previous positive culture for *S. aureus*, no hospitalization or surgery in the last 12 months, and no invasive devices. Infections that did not meet these criteria were considered HA (7).

Infection sites were classified by the attending clinicians as skin/soft tissue, osteoarticular, primary bloodstream infection, respiratory tract, central nervous system, ventriculitis, intra-abdominal, upper airway, urinary tract, surgical site, or endocarditis according to the clinical picture and complementary exams. Patients with secondary bacteremia were classified according to the primary site of infection. To be considered a surgical site infection, it must have occurred within 30 days of the procedure or within 90 days for surgeries involving prosthetic material.

### Antimicrobial susceptibility test

The antimicrobial susceptibility profile was determined using the disk-diffusion technique for the following antibiotics: oxacillin, gentamicin, trimethoprim-sulfamethoxazole (TMP-SMX), clindamycin, doxycycline, rifampicin, and ciprofloxacin. Vancomycin susceptibility was determined using ETest strips. The results were interpreted according to Clinical and Laboratory Standards Institute definitions (8).

### Data collection and analysis

In addition to the primary infection site and antimicrobial susceptibility test results, sex and age data were collected. Infections were classified as CA or HA and attributed to MSSA or MRSA, according to the criteria mentioned above.

This study was approved by the hospital's research ethics committee (37140820.1.0000.5683).

All results were expressed as absolute and relative frequencies (percentages) for categorical variables or measures of central tendency and dispersion (mean, standard deviation, median, and variation range) for numeric variables.

For asymmetrically distributed continuous variables, the Mann–Whitney test was used to compare the groups. For categorical data, Pearson's *χ*^2^ test was used in conjunction with analysis of the adjusted residuals. Linear trend *χ*^2^ testing was used to analyze evolutionary rates. *p* < 0.05 was considered statistically significant. The statistical analyses were performed in IBM SPSS Statistics 27.0 (IBM, Armonk, NY, USA).

## Results

Of the 654 cultures evaluated, 91 were excluded due to contamination (*N* = 44, 48.3%), polymicrobial growth (*N* = 41, 45.1%), and incomplete medical records (*N* = 6, 6.6%). A total of 563 patients were included and, among patients with a CA infection, the MRSA prevalence was 46.1% (129/280), whereas only 8.1% of HA infections involved MRSA isolates (23/283). The sample was 56% male and its median age was 2 years. There was no significant age difference between patients with CA-MSSA and CA-MRSA infections (*p* = 0.928). The majority of collected materials were abscess secretions (50.8%) and blood cultures (41.6%). The most frequent primary infection site was the skin/soft tissues (38.9%), followed by primary bloodstream (23.6%) and surgical site (20.8%) (Table 1).

**Table 1:** Sample characterization.

Variables	Overall sample (*n* = 563; 100%)	Community-acquired (*n* = 280; 49.7%)	Healthcare-associated (*n* = 283; 50.3%)
Age (years) – median (min-max)	2 (0–17)	3 (0–17)	1 (0–17)
Male – *n* (%)	315 (56.0)	150 (53.6)	165 (58.3)
Material – *n* (%)			
Abscess secretion	286 (50.8)	200 (71.4)	86 (30.4)
Blood	234 (41.6)	66 (23.6)	168 (59.4)
Cerebrospinal fluid	17 (3.0)	0 (0.0)	17 (6.0)
Peritoneal fluid	7 (1.2)	0 (0.0)	7 (2.5)
Pleural effusion	15 (2.7)	13 (4.6)	2 (0.7)
Other	4 (0.7)	1 (0.4)	3 (1.1)
Primary site – *n* (%)			
Skin/soft tissue	219 (38.9)	201 (71.8)	18 (6.4)
Osteoarticular	38 (6.7)	34 (12.1)	4 (1.4)
pBSI	133 (23.6)	11 (3.9)	122 (43.1)
Respiratory	30 (5.3)	24 (8.6)	6 (2.1)
CNS/ventriculitis	5 (0.9)	0 (0.0)	5 (1.8)
Upper airways	4 (0.7)	4 (1.4)	0 (0.0)
Urinary tract	1 (0.2)	1 (0.4)	0 (0.0)
Intra-abdominal	15 (2.7)	4 (1.4)	11 (3.9)
Surgical site	117 (20.8)	0 (0.0)	117 (41.3)
Endocarditis	1 (0.2)	1 (0.4)	0 (0.0)
Phenotype – *n* (%)			
MSSA	411 (73.0)	151 (53.9)	260 (91.9)
MRSA	152 (27.0)	129 (46.1)	23 (8.1)

CNS, central nervous system; pBSI, primary bloodstream infection.

**Figure 1:**
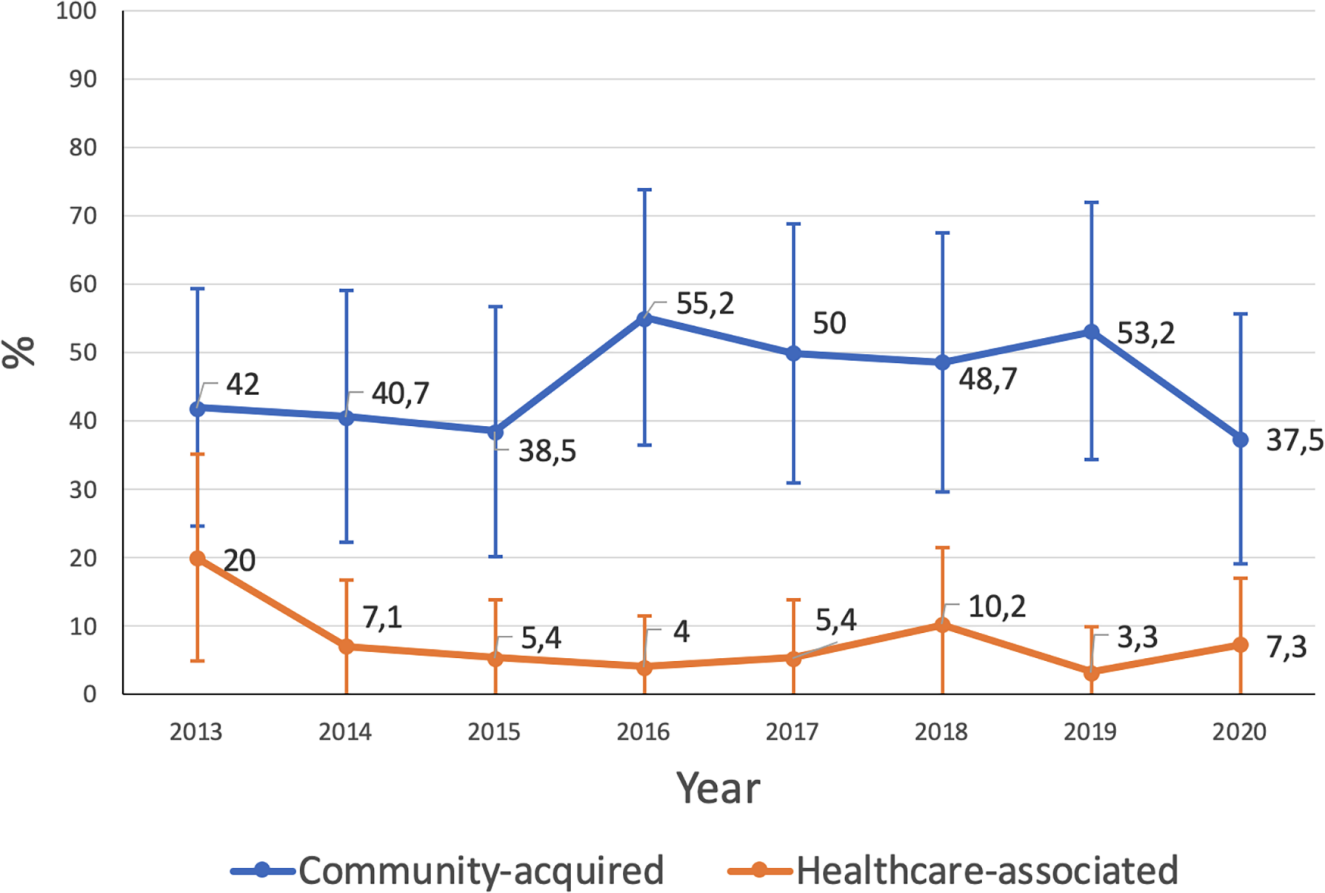
Proportion of MRSA among all staphylococcal infections.

Regarding the strains' oxacillin susceptibility (MSSA or MRSA), 46.1% of CA infections were MRSA and 8.1% of HA infections were MRSA. The MRSA prevalence did not change significantly during the study period for CA or HA infections (*p* = 0.515 and *p* = 0.137, respectively) (Table 1 and Figure 1).

There was a significant association between primary infection site and phenotype. Among CA infections, MSSA was significantly more frequent in osteoarticular and primary bloodstream infections (*p* = 0.001). On the other hand, MRSA was significantly more frequent in respiratory and intra-abdominal infections (*p* = 0.001). In HA infections, MSSA was significantly more frequent in primary bloodstream infections, while MRSA was significantly more frequent in skin/soft tissue and respiratory infections (*p* = 0.001) (Table 2).

**Table 2:** Association between primary infection site and phenotype in each patient group.

Primary site	Community-acquired			Healthcare-associated			Total *n* = 563 *n*
	MSSA (*n* = 151) *n* (%)	MRSA (*n* = 129) *n* (%)	TOTAL (*n* = 280) *n* (%)	MSSA (*n* = 260) *n* (%)	MRSA (*n* = 23) *n* (%)	TOTAL (*n* = 283) *n* (%)	
Skin/soft tissue	106 (52.7)	95 (47.3)	201 (100)	14 (77.8)	4 (22.2)*	18 (100)	219
Osteoarticular	25 (73.5)*	9 (26.5)	34 (100)	4 (100)	0 (0.0)	4 (100)	38
pBSI	10 (90.9)*	1 (9.1)	11 (100)	117 (95.9)*	5 (4.1)	122 (100)	133
Respiratory	6 (25.0)	18 (75.0)*	24 (100)	3 (50.0)	3 (50.0)*	6 (100)	30
CNS/ventriculitis	0 (0.0)	0 (0.0)	0 (0.0)	5 (100)	0 (0.0)	5 (100)	5
Upper airways	2 (50.0)	2 (50.0)	4 (100)	0 (0.0)	0 (0.0)	0 (0.0)	4
Urinary tract	1 (100)	0 (0.0)	1 (100)	0 (0.0)	0 (0.0)	0 (0.0)	1
Intra-abdominal	0 (0.0)	4 (100)*	4 (100)	10 (90.9)	1 (9.1)	11 (100)	15
Surgical site	0 (0.0)	0 (0.0)	0 (0.0)	107 (91.5)	10 (8.5)	117 (100)	117
Endocarditis	1 (100)	0 (0.0)	1 (100)	0 (0.0)	0 (0.0)	0 (0.0)	1

CNS, central nervous system; pBSI, primary bloodstream infection.

* Significant difference (*p* < 0.05) between the phenotypes (MSSA and MRSA) at the primary sites within each group (CA or HA).

**Figure 2:**
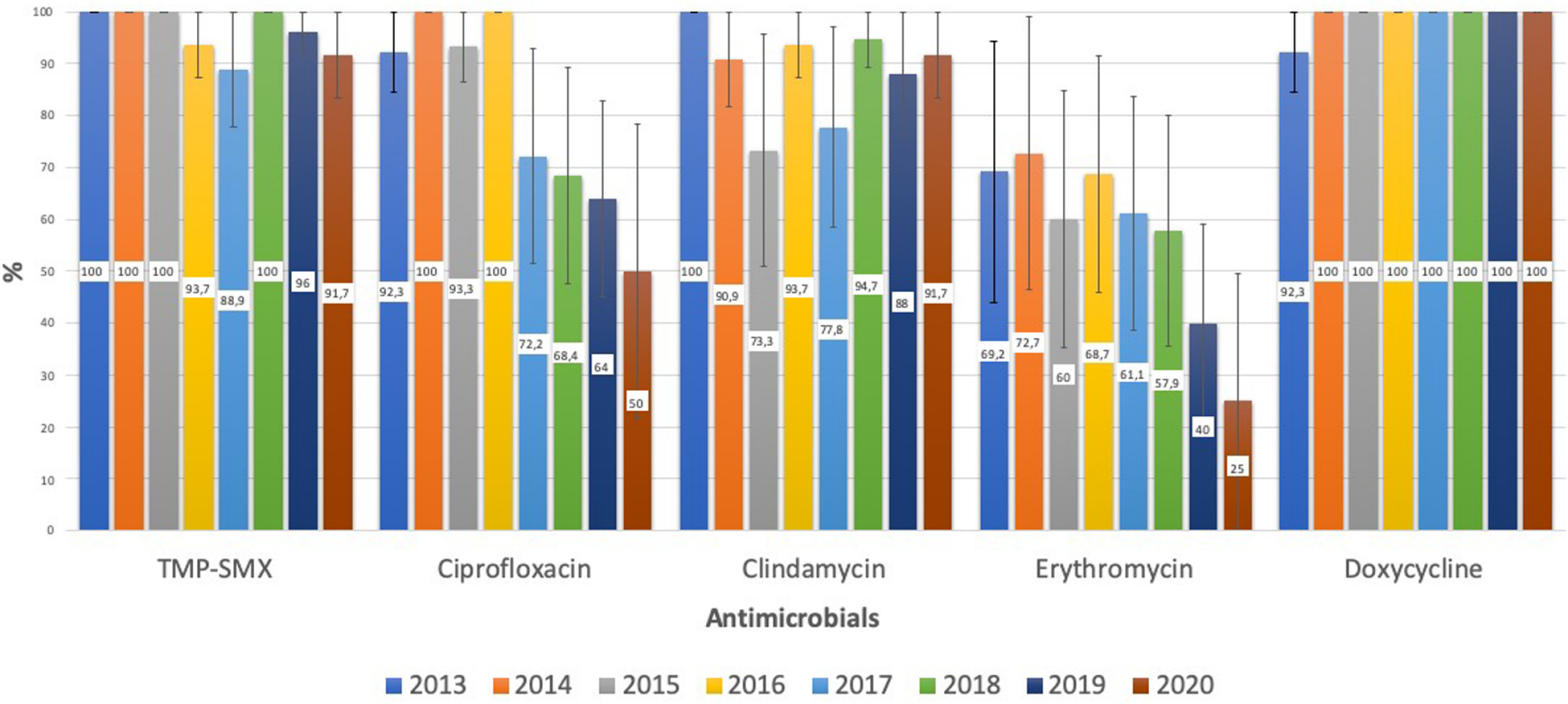
CA-MRSA: evolution of antimicrobial susceptibility. TMP-SMX, trimethoprim-sulfamethoxazole.

Regarding the susceptibility of *S. aureus* to the other antimicrobials, CA-MSSA had a high rate of susceptibility to TMP-SMX, doxycycline, and ciprofloxacin, and a low rate of susceptibility to clindamycin and erythromycin (64.9% and 58.3%, respectively), with a downward but non-significant (*p* = 0.056) trend toward clindamycin susceptibility during the study period. CA-MRSA had a low rate of susceptibility to ciprofloxacin and erythromycin (78.3% and 55.8%, respectively) and these rates significantly declined over the study period (*p* < 0.001 and *p* = 0.005, respectively). The susceptibility of CA-MRSA to TMP-SMX, clindamycin, and doxycycline remained high, being 96.1%, 88.4% and 99%, respectively (Figure 2).

Regarding HA infections, MSSA had a low susceptibility rate to clindamycin and erythromycin (70.8% and 66.2%, respectively). MRSA had a low rate of susceptibility to ciprofloxacin, clindamycin, and erythromycin (52.2%, 60.9%, and 34.8%, respectively). HA-MRSA remained highly susceptible to TMP-SMX and doxycycline (91.3% and 95%, respectively).

Vancomycin resistance was not observed in any of the samples, and only 4 isolates showed resistance to rifampicin: 3 from CA infections (2 MSSA and 1 MRSA) and 1 from a HA infection (MSSA). All isolates were susceptible to some other antimicrobial in addition to vancomycin.

## Discussion

In Brazil, few studies with adequate sample sizes have attempted to determine the prevalence of MRSA in CA infections, which increases the importance of the present study. Our results include alarming data on high resistance to oxacillin, the main antibiotic used to treat clinical community-acquired staphylococcal syndromes, reaching a mean of 46.1% for community-acquired isolates. The chance of therapeutic failure with oxacillin or another beta-lactam in these infections is high, which reinforces the importance of submitting clinical specimens to culture. The pediatric literature recommends an association of beta-lactam and vancomycin for invasive Staphylococcus infections, which is to be adjusted after determining susceptibility, but this practice does not appear to be widespread in pediatric services (9–11).

In contrast to high resistance in community strains, we observed a low prevalence of MRSA (8.1%) in HA infections, indicating that MRSA has been losing ground among nosocomial infections and gaining importance in CA infections. Thus, the choice of antibiotic must be adapted to local epidemiology, with anti-MRSA agents included in the initial regimen for CA infections.

Clindamycin is the main recommendation for CA staphylococcal infections when resistance is suspected, especially in regions with a high prevalence of CA-MRSA (10, 11). However, our study shows a high prevalence (35.1%) of clindamycin resistance among CA-MSSA, with the resistance rate falling to 11.6% in CA-MRSA. These data should raise concern about the empirical use of clindamycin, mainly in cases of MSSA. In comparison, the resistance rates for trimethoprim-sulfamethoxazole and doxycycline were very low: 6.6% (MSSA) and 3.9% (CA-MRSA) for TMP-SMX, and 1.6% (MSSA) and 1% (CA-MRSA) for doxycycline, making them interesting therapeutic options for non-invasive infections. Doxycycline's use has been previously limited to children 8 years and older due to dental toxicity concerns but more recent data support the recommendation by the American Academy of Pediatrics that doxycycline can be safely used for short durations (21 days or less) regardless of the patient's age (9).

The changes in MRSA rates in 2020, which did not follow the pattern of previous years, coincide with the onset of the non-pharmaceutical measures implemented to reduce Sars-CoV-2 circulation (i.e., social isolation and school and daycare centre closures), which could have impacted the dynamics of pediatric CA-MRSA transmission and hospitalization, making a more accurate interpretation difficult.

Our study has several limitations. First, it was a retrospective, single-center study. Samples of non-sterile materials, such as respiratory secretions, were excluded due to the difficulty in differentiating between infection, colonization, and contamination. This, for example, could have led to an underestimation of respiratory infections, both for CA staphylococcal pneumonia and ventilator-associated pneumonia. Another limitation was that we analyzed cases based on positive culture for *S. aureus*. Excluding staphylococcal infections without a positive culture may have resulted in selection of more serious cases. This is especially important, considering that CA-MRSA strains can result in suppurative and more severe infections due to the production of toxins such as Panton-Valentine leukocidin. We believe that this, in association with the majority of CA infections being skin/soft tissue infections (71.8%), may have led to overestimation of the MRSA rate in CA infections. Nevertheless, the rate we found was significantly high, especially compared to similar studies in Brazil (1, 12).

In conclusion, our study showed high rates of MRSA in CA staphylococcal infections, highlighting the need to review, according to local epidemiology, initial treatment protocols for severe staphylococcal infections that require hospitalization. Further epidemiological studies are also needed to determine susceptibility differences in each region of the world.

## Data Availability

The raw data supporting the conclusions of this article will be made available by the authors, without undue reservation.
